# Same Sentences, Different Grammars, Different Brain Responses?: An MEG Study on Case and Agreement Encoding in Hindi and Nepali Split-Ergative Structures

**DOI:** 10.1162/NOL.a.247

**Published:** 2026-06-04

**Authors:** Dustin A. Chacón, Subhekshya Shrestha, Brian W. Dillon, Rajesh Bhatt, Diogo Almeida, Alec Marantz

**Affiliations:** University of California, Santa Cruz, Santa Cruz, CA, USA; New York University Abu Dhabi, Abu Dhabi, United Arab Emirates; University of Massachusetts Amherst, Amherst, MA, USA; New York University, New York, NY, USA

**Keywords:** agreement, case, electrophysiology, ergativity, Hindi, magnetoencephalography (MEG), Nepali, neurolinguistics, psycholinguistics, sentence processing

## Abstract

At first glance, the brain’s language network appears to be universal, but languages clearly differ. Does the brain adapt to the specific details of individual grammatical systems? Here, we present a magnetoencephalography (MEG) study on case and agreement in Hindi and Nepali. Both languages use split-ergative case systems. However, these systems interact with verb agreement differently—in Hindi, case features conspire to determine which noun phrase (NP) the verb agrees with (subject, object, or neither), but in Nepali the verb always agrees with the subject NP. We found that NPs with different case values elicit different MEG signals around 200–500 and 600–900 ms. In subsequent exploratory analyses, we failed to find a reliable difference in this brain activity between the two languages corresponding to the different relations between case and agreement. However, we identified a portion of the left temporoparietal junction as exhibiting a statistically nonsignificant effect that may warrant further investigation.

## INTRODUCTION

Languages can appear quite different from one another. Nonetheless, a typical human can learn and use any natural human language. The organization of the brain’s “language network” appears to be largely uniform across languages, despite the diversity in features they may have or the kinds of information their structures encode (Dunagan et al., 2022; Malik-Moraleda et al., 2022), although some results suggest some differences (Wei et al., 2023). Given the reasonable assumption that neural structures and language processing strategies are essentially the same across languages, differences in neural activity should reflect the way that a uniform representational and processing system deals with parametric differences between grammatical systems. For example, a predictive processing mechanism will predict the arguments of a verb from the verb in a subject–verb–object (SVO) language, whereas the verb might be predicted from both arguments in a subject–object–verb (SOV) language, or languages with strict word order and no case marking may weight word order as a stronger predictive cue than languages with freer word order and case marking (e.g., Schelesewsky & Bornkessel-Schlesewsky, 2009). Identifying systematic differences in neural activity in language processing between languages can serve as a key strategy for relating grammatical descriptions, processing mechanisms, and neurobiology.

We examine this question through the lens of case and agreement dependencies. Case morphology, such as the difference between nominative *he* and accusative *him*, provides the comprehender with important cues to “who did what to whom” via syntactic relations. Like case, agreement also guides the comprehender in identifying entities in the sentence. In English, the verb agrees (corresponds in form) with the subject noun phrase (NP) in number—*the boy quickly run-**s**, the boy-**s** quickly run*. However, different languages require that the verb relates to its arguments differently. For instance, in many languages, such as Hindi, Nepali, Arabic, or Hebrew, the verb agrees with its subject in gender, unlike English—*the boy quickly run-**s**, the girl quickly run-**s***. Thus, language processing entails attending to different features at different times in different languages.

Here, we leverage the “split-ergative” agreement systems of two very typologically similar languages: Hindi and Nepali. Hindi and Nepali are both predominantly SOV languages from the Indo-Aryan language family. In both languages, the subject NP requires an ergative case suffix (ने -*ne* in Hindi, ले -*le* in Nepali) in simple past (perfective) sentences, but not in the simple (imperfective) present, imperfective past, or future. In the simple present, the verb agrees with the nominative subject in number, gender, and person, much like Western European languages. However, in the perfective past, the languages use different rules. In Hindi, the verb usually agrees with the object in the simple past and not with the subject carrying the ergative ने -*ne* suffix. In Nepali, the verb also agrees with the subject carrying the ले -*le* suffix in the past tense, i.e., no difference is observed between present and past tense in Nepali with respect to agreement.

These grammatical features mean that different information is accessible at different points in Hindi and Nepali in sentences that are otherwise structurally similar. In Nepali, comprehenders can identify the expected number, gender, and person of the verb shortly after encountering the subject NP, regardless of its case. However, in Hindi, the comprehender’s expectations of the verb’s form differ depending on the presence of the ergative suffix ने -*ne*. If the subject NP is bare, then Hindi comprehenders know that the verb will match with the subject NP in number, person, and gender, just as in Nepali. If the subject NP has the ergative suffix ने -*ne*, then the comprehender must wait to encounter the object NP to determine the verb’s features. In other words, Nepali comprehenders can always identify the subject NP as the “agreement controller,” i.e., the noun with which the verb must match in features. By contrast, Hindi comprehenders must examine the morphology of the subject NP and object NP to determine which NP is the controller and may not know the likely agreement specification on the verb until after integrating both NPs into the parse of the sentence.

Here, we investigate the relation between brain activity and the different accessibility of agreement-relevant cues during sentences processing in Hindi and Nepali using magnetoencephalography (MEG). We focus on the evoked brain activity during processing of the object NP, prior to the verb, in simple SOV sentences in ergative-perfective structures and nominative-imperfective structures, focusing on NP–verb agreement in gender marking. We assume that Hindi comprehenders explicitly identify and store in memory an argument NP as the agreement controller (Bhatia, 2019; Bhatia & Dillon, 2022), which is not coextensive with a specific grammatical role (subject, object) or case morphosyntax (e.g., nominative). This is consistent with the view that agreement processing depends on anticipatorily encoding features of the verb, including agreement features such as gender, prior to encountering it (Chacón, 2022; Eberhard et al., 2005; Keshev et al., 2025; Keshev & Meltzer-Asscher, 2024). Due to the agreement rules of Hindi, the expected features of the verb cannot be uniformly identified until the object NP, i.e., after the case features of both principal arguments have been identified and integrated into a syntactic structure. By contrast, Nepali comprehenders should be able to identify the likely morphosyntactic features on the verb shortly after processing the subject NP, and thus, these predictive processes do not need to be present during the processing of the object NP.

The neuroanatomical correlates of the processes we investigate here are not well understood. We have two key hypotheses. The first hypothesis is that subject and object case morphology will interact in the brains of Hindi comprehenders during the processing of an object NP. Specifically, we expect that the processing of a bare object NP that controls agreement vs. the processing of a bare object NP that does not will show different patterns of activity, despite occupying identical positions in the syntactic structure, having the same lexical semantics, and having the same thematic relation to the verb. Importantly, we hypothesize that this difference in activity should not be observed in Nepali, nor for accusative-marked object NPs in constructions in either language, as these NPs do not control agreement in either language. We do not have a priori hypotheses about the directionality of this effect, nor the region that this will occur in, although we suspected that it would be in a key area associated with case agreement processing: left prefrontal regions, left anterior temporal regions, or inferior parietal regions.

**Figure 1.**
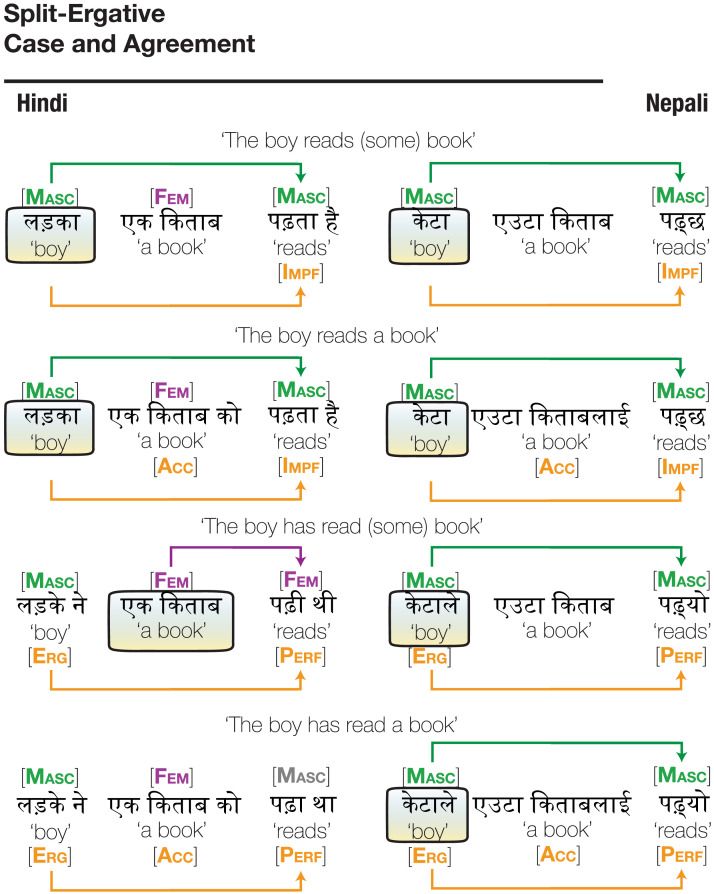
Illustration of the split-ergative case systems and agreement systems in Hindi and Nepali. In Hindi, unmarked (nominative) subject NPs imply imperfective verb aspect, and control agreement with the verb. Ergative subject NPs imply perfective verb aspect and object agreement when the subsequent object NP is bare. In Nepali, nominative subject NPs imply imperfective verb aspect, whereas ergative subject NPs imply perfective verb aspect. Subject NPs are always the controller of agreement. Agreement controllers are in yellow boxes.

Crucially, we make no a priori hypotheses about main effects of ergative vs. nominative case during the processing of the subject NP, nor the processing of the accusative vs. bare case during the processing of the object NP. There are likely many differences engendered by these features in these time windows, and instead we focus our hypothesis on the interaction of these features during the processing of the object NP period, since this is where Hindi comprehenders should have accumulated sufficient evidence to identify the inflectional features on the upcoming verb (Figure 1).

### Case-and-Agreement Processing and Predictive Encoding

Subject–verb agreement dependencies are extensively studied in psycholinguistics as a window into the memory operations that support language comprehension generally (Badecker & Kumiak, 2007; Dillon et al., 2013; Nicol et al., 1997; Wagers et al., 2009) and as a window into the kinds of representations that are built in real time (Bhatia & Dillon, 2022; Bock & Miller, 1991; Chacón, 2022; Franck et al., 2002). Subject–verb agreement processing likely consists of at least two distinct stages; the first involves identifying possible agreement controllers and predicting likely morphosyntactic features of the upcoming verb (Bhatia & Dillon, 2022; Chacón, 2022; Eberhard et al., 2005; Keshev et al., 2025; Wagers et al., 2009), while the second involves retrieval of the subject NP’s features after the verb is encountered (Badecker & Kumiak, 2007; Dillon et al., 2013; Nicol et al., 1997; Wagers et al., 2009) using a cue-based retrieval mechanism (Badecker & Kumiak, 2007; Lewis & Vasishth, 2005; Lewis et al., 2006; Wagers et al., 2009).

Electroencephalography (EEG) recordings demonstrate rapid detection of verbs that satisfy vs. violate agreement rules (Bhattamishra et al., 2022; Choudhary et al., 2009; Friederici, 2002; Hagoort et al., 1993; Molinaro et al., 2011; Nevins et al., 2007). Agreement violations, in which the verb and its argument mismatch in a feature, usually elicit a biphasic response, with a left anterior negativity (LAN) ∼300 ms post–verb onset, followed by a P600, a positive deflection ∼600 ms post–verb onset. These earlier responses may reflect rapid detection of a violated predicted verb form, and later responses may reflect control processes needed to revise the interpretation of the sentence (Molinaro et al., 2011). Similarly, using MEG, Tucker et al. (2014) found greater activity for verbs that satisfied agreement rules than for ungrammatical verbs that mismatched the subject in number in the left posterior superior temporal lobe (LpSTL), ∼700 ms post–verb onset.

Hemodynamic measures investigating agreement violations have implicated a variety of brain regions in agreement configurations, including the left inferior frontal gyrus (LIFG), portions of the left anterior temporal lobe (LATL), left posterior temporal lobe (LPTL), and the left and right temporal parietal junction (LTPJ and RTPJ, respectively). In a functional magnetic resonance imaging (fMRI) study investigating case and agreement violations in Basque, a language with ergative case and both obligatory subject–verb and object–verb agreement, Nieuwland et al. (2012) observed greater activation in LTPJ and RTPJ for object–verb agreement violations. They also found greater activation for object–verb mismatch in the bilateral middle frontal gyri (MFG). In an fMRI study in Spanish, a language with obligatory gender and number agreement between nouns and modifiers, Carreiras et al. (2015) found greater activity in LIFG for adjective–noun and determiner–noun pairs that mismatched in gender or number. They additionally found greater activation in right parietal areas for determiner–noun and adjective–noun pairs that mismatched in number (see also Carreiras, Duñabeitia, et al., 2010). Similarly, in an fMRI study in Spanish, Quiñones et al. (2014) identified greater activity in LTPJ and left MFG (LMFG) regions for ungrammatical subject NP–verb agreement. They also identified greater activity in LATL, LpSTL, LIFG, and portions of the left orbitofrontal cortex (OF) for grammatical structures that exceptionally allow for a mismatch in person features (“unagreement”). Quiñones et al. (2018) found that grammatical nouns with transparent gender marking (*libr-**o*** “book”; -*o* is the masculine gender marker) engaged LPTL and LTPJ and the right homologs more than “opaque” nouns (*lápiz* “pencil”), but opaque nouns resulted in greater activity in bilateral frontal and superior temporal lobe. Finally, in an fMRI study in Japanese, Cui et al. (2022) found different patterns of activity for verb marking corresponding to the relative social status of the speaker vs. other NP referents in left frontal regions, LATL, and LTPJ.

A similar broad network of left perisylvian regions is implicated in production of inflected forms. In direct neuron recordings, Sahin et al. (2006) found greater activity in the LIFG when participants produced English inflected verb and noun forms ∼300 ms after word onset (*rock-s*, *rock-ed*) compared to uninflected forms. Similar findings were observed using production study using fMRI by Kielar et al. (2011). Using a similar paradigm in MEG with word production in English, Hauptman et al. (2022) observed greater activity in LIFG and LATL corresponding to verb and noun inflection, approximately 300 ms after the cue to begin speaking was provided.

However, due to the limitations of these methodologies, it has been difficult to draw firm inferences about which brain areas are responsible for which computations. EEG recordings have poor spatial resolution, meaning it is difficult to identify the neural generators for the measured activity. Inversely, hemodynamic measures afford poorer temporal resolution, rendering it difficult to disentangle memory retrieval operations encountered at the verb versus preverbal computations supporting selection of agreement controllers and preemptive encoding of verb features. Finally, although direct neuron recordings and MEG recordings enjoy high spatial and temporal resolution, these studies have predominantly focused on computations executed at the verb, rather than anticipatory processes that might occur before the verb is encountered, e.g., identifying a controller and predicting the verb’s features. Thus, little is understood about how the brain executes these computations. Nonetheless, these studies demonstrate that LIFG, LATL, and LTPJ are likely candidates in processing argument–verb agreement relations.

The hypothesis that we entertain in this article is that comprehenders of Hindi exploit morphosyntactic case marking as a cue for predicting agreement features and that these processes are different in Nepali. Overwhelmingly, comprehenders appear capable of integrating many cues to predict upcoming semantic and syntactic features of an upcoming verb (cf. Ferreira & Qiu, 2021). In EEG recordings, a greater negative amplitude around 200–400 ms, the “N400,” is associated with less predictability of a word given its prior context (*I drink my coffee with sugar and socks/cream*) (Kutas & Federmeier, 2011; Kutas & Hillyard, 1980). The MEG correlate of the N400, the “M350,” and fMRI responses to unexpected words localize the effect of responses to unpredictable words in the left middle temporal gyrus (LMTG), although left frontal and left OF regions are also crucial for detecting unpredicted words (Baumgaertner et al., 2002; Halgren et al., 2002; Lau et al., 2008; Lau & Namyst, 2019; Pylkkänen et al., 2004; Van Petten & Luka, 2006).

Given that comprehenders are capable of integrating many cues into their predictions, then comprehenders of different languages may attend to different cues depending on the structural features of their languages. This has been demonstrated robustly over a series of studies by Bornkessel-Schlesewsky and colleagues (Bornkessel-Schlesewsky et al., 2011; Bornkessel-Schlesewsky & Schlesewsky, 2008, 2009, 2016). For instance, they argue that users of German, which exhibits flexible word order and case marking, rely on case information and animacy more than word order to guide their interpretation of NPs (Schelesewsky & Bornkessel-Schlesewsky, 2009). In an fMRI study in German, a language with freer word order and case marking, Bornkessel et al. (2005) found greater activity for sentences with unlikely word order and verb combinations compared to more likely combinations in left frontal regions, LpSTL, and left ventral premotor cortex. Additionally, they found that German NPs with ambiguous case (*Lehrerinnen* “teachers,” which may be dative or nominative) modulate neural activity differently in LTPJ and LpSTL than NPs with unambiguous case marking (*den*_[Dat]_ *Lehrerinnen*_[Dat]_ “(to) the teachers”). Mandarin Chinese, by contrast to German, is a language that overwhelmingly prefers SVO word order, and features no case or agreement morphology. Mandarin Chinese users therefore heavily rely on word order and animacy as cues to interpreting sentences (Wang et al., 2009, 2012), although this too can be modulated by the demands of grammatical constructions (e.g., “adversative passives” in Philipp et al., 2008). Similarly, Gattei et al. (2015) found that in Spanish, a language with flexible word order like German but with less explicit case morphology, comprehenders used both word order and case marking to infer the likely semantic class of the verb in “marked” word order-case configurations that are highly associated with particular verbal semantics, suggesting complex top-down knowledge of grammatical properties and statistical regularities guide predictive processing (see also Simonsen & Chacón, 2025).

The majority of work on prediction in language comprehension contrasts the neural responses to predicted vs. unpredicted material. What do we know about the mechanisms of predictions themselves? In an MEG study on two-word adjective–NPs in English with highly predictive adjectives (e.g., *stainless* strongly predicts *steel*), Fruchter et al. (2015) found that the predictiveness of adjectives (e.g., *p*(“steel” | “stainless”)) correlated with MEG activity in LATL regions 100–500 ms post–adjective onset and LIFG regions 400–500 ms post–adjective onset. Similarly, Wang and colleagues also examined the neural activity prior to a predicted word to determine the neural correlates of committing to a predicted word. They compared the neural activity in EEG and MEG recordings after highly predictive verbs vs. less predictive verbs in Mandarin Chinese (Wang et al., 2018) and English (Wang et al., 2020). Using a representational similarity analysis (Kriegeskorte et al., 2008), they found greater similarity in the spatial patterns of the evoked responses 400–600 ms after highly constraining verbs compared to less constraining verbs, before the predicted noun was encountered.

Our prediction specifically concerns the early encoding or predicting of agreement features of the verb given the case marking on the preceding NPs. But, what are the neuroanatomical correlates of the processes implicated in processing case? As mentioned earlier, fMRI studies in German and Basque point to LpSTL, bilateral TPJ, bilateral frontal regions as key regions. Additionally, in an fMRI study in English, Yokoyama et al. (2012) found greater activation for left frontal regions, LTPJ, and LPTL when participants were instructed to select the appropriate case form in a sentence context. Thus, there appears to be significant overlap between the neuroanatomical regions implicated in agreement processing (LIFG, LATL, LTPJ), case processing (LTPJ, LIFG), and lexical prediction (LPTL, LATL).

### Split-Ergativity in Hindi and Nepali

Our study focuses on Standard Hindi and Nepali. Both languages are typically written in the Devanagari script and use SOV word order. Hindi nouns, verbs, and adjectives are marked for masculine or feminine gender (typically with -*ā* masculine singular, -*e* for masculine plural, and -*ī* for feminine). Nepali animate nouns, verbs, and adjectives are marked for gender (typically -*ā* and -*o* for masculine and -*ī* and -*i(n)* for feminine), and verbs inflect for number. Inanimate nouns typically default to masculine in Nepali.

Both languages use a split-ergative case system. In the perfective aspect, the subject NP normally must end in the ergative case suffix ने -*ne* in Hindi and ले -*le* in Nepali. In other tense/aspect combinations, the subject NP normally appears in the nominative case, which is not associated with a suffix. For concreteness, we refer to the argument of intransitive predicates and the agentive or external argument of transitive predicates as “the subject NP,” regardless of case and agreement, and we refer to the theme, patient, or internal argument of a transitive predicate as “the object NP,” although these labels can be problematic for some ergative languages (Aldridge, 2008; Dixon, 1994; see also Carreiras, Carr, et al., 2010; Laka, 2012, for relevance to language processing with respect to Basque; Polinsky et al., 2012, for Avar; and Logenbaugh & Polinsky, 2016; Tollan et al., 2019, for Niuean). Unlike in other languages, ergative subjects in Hindi and Nepali exhibit more of the “typical” properties of subjects compared to their nominative counterparts (e.g., de Hoop & Narasimhan, 2009, for Hindi).

Although the correlation between aspect and subject case morphology is strong, it is not absolute in either language. In Hindi, the ergative suffix is optional with some intransitive verbs in the perfective aspect (such as खाँस - *khāṃs* - “cough”), i.e., even if there is only one NP argument. Some so-called “light verbs,” verbal auxiliaries that combine with other verbs to modulate their aspectual interpretation, require the assignment of ergative case, and others exceptionally require that the subject is bare, even for transitive predicates in the perfective aspect (Kachru, 1980; Mahajan, 2012). Lastly, Hindi users may choose to mark the agent with ergative case to assert that the event was intentional or may choose to omit it to express a nonvolitional action in some contexts (Butt & Deo, 2017). In Nepali, the ergative suffix ले -*le* may also appear with verbs that are not perfective due to an array of factors (see Li, 2007, for an overview). Nepali users may use the ergative suffix to disambiguate whether an inanimate or non-human NP is the agent of the action (Abadie, 1974), to focus or “emphasize” the subject (Bickel, 2011), and to modulate the relationship between the subject and predicate, i.e., to express an individual-level predicate (Butt & Poudel, 2007) or to express a “categorical proposition” (Kuroda, 1972; Lindemann, 2019). Unlike in Hindi, light verbs are reported to not affect the case morphology of the subject (Slade, 2014). Despite these complications, EEG evidence from Hindi demonstrates that ergative case morphology is rapidly integrated as a predictive cue for perfective aspect. For instance, Choudhary et al. (2009) found that evoked responses at the verb exhibited increased N400 and P600 amplitudes for imperfective verbs occurring after an ergative subject and an increased N400 amplitude for perfective verbs occurring after a nominative subject. Similarly, Mathew et al. (2026) reported that subject NP case morphology is a predictive cue for transitivity and light verb choice in Hindi, given the interaction between these elements.

In addition to this subject NP case alternation, both languages also exhibit an object NP case alternation. Animate object NPs obligatorily surface with the accusative/dative case suffix को -*ko* in Hindi and लाई -*lāī* in Nepali. Inanimate object NPs may occur bare or with the accusative case. Inanimate object NPs that are marked with the accusative case carry a specific or definite interpretation. The variations in case of the subject NP and object NP are orthogonal and do not appear to change their thematic interpretation, syntactic position, or the grammatical relations between the NP arguments and the verb apart from agreement.

Hindi verb agreement is descriptively governed by what Bhatia (2019) calls the Hindi Agreement Generalization, based on Pandharipande and Kachru (1977): The verb agrees with the most structurally prominent NP that is unmarked for case. This results in an interaction between ergativity/aspect marking and agreement. In sentences in the imperfective aspect (e.g., simple present), the subject NP typically does not end in ergative suffix ने -*ne*. The subject NP controls agreement in this configuration, since it is the highest “bare” NP. In the perfective aspect, the subject must typically be marked with the ergative suffix ने -*ne*. In these contexts, the subject NP is blocked from controlling agreement. Instead, the next prominent NP must be considered. If the object NP is bare, then it controls agreement, since it is now the most prominent NP without case. However, if the object NP is marked with accusative case को -*ko* to mark animacy or specificity, then the object NP is also “blocked” from agreement. If both subject and object NPs are marked with case, then the verb must surface as a third person, singular, masculine “default” form. In Nepali, by contrast, verbs always agree with subject NP, regardless of whether it is ergative or bare. More exhaustive studies on case and agreement in Hindi can be found in Butt and King (2004), Bhatt (2005), and Mahajan (2017).

Previous studies on the processing of Hindi/Urdu using EEG have revealed a similar rapid sensitivity to agreement violations as observed in other languages. In an agreement violation paradigm, Nevins et al. (2007) found that subject–verb agreement violations produced a greater P600 response compared to grammatical controls. This was observed for verbs that mismatched in number, gender, and person with the subject NP, with the greatest amplitude difference corresponding to person + gender mismatches. In a study leveraging the correspondence between subject NP case and verb tense/aspect, Choudhary et al. (2009) found distinct event-related potential (ERP) patterns to verbs that mismatch in aspect with the expected case marker. Bare subject NPs followed by an ungrammatical simple perfective verb produced greater N400 amplitude post–verb onset compared to grammatical controls, and ergative subject NPs followed by an ungrammatical imperfective verb produced both greater N400 and P600 responses post–verb onset (see also Bickel et al., 2015). Bhattamishra et al. (2022) found an increase in P600 amplitude for gender agreement errors for animate subject NPs, but a negativity observed in posterior midline sensors for gender agreement with inanimate subject NPs (see also Gulati & Choudhary, 2023, and Gulati et al., 2024, for different results in Punjabi). Studies on Basque, another split-ergative language in which verbs agree with both subject and object NPs, reveal similar rapid detection of agreement violations for both subject and object agreement violations (Díaz et al., 2016; Zawizseski & Friederici, 2009; but see Chow et al., 2018).

Moreover, the formal relationship between a verb and its arguments affects early stages of sentence production. In an EEG study, Sauppe et al. (2021) asked participants to spontaneously produce sentences describing pictures in a cued tense/aspect. The pictures were designed to elicit both intransitive verbs and transitive verbs. In one condition, participants were cued to use the perfective aspect, which requires the ergative case suffix ने -*ne* for the transitive verbs, but not the intransitive verbs. In the other condition, the verbs were in the imperfective, and thus both subject NPs were in the nominative form. Shortly after the onset of picture, differences in alpha wave activity were observed between marked (ergative) and unmarked (nominative) forms. Differences in theta wave activity were observed in conditions in which participants produced sentences with the same subject NP case marking vs. distinct subject NP case marking, 100–300 ms post–stimulus onset in left anterior sensors.

In sentence comprehension, behavioral psycholinguistic findings also demonstrate that users of Hindi use subject NP and object NPs to predict features of the verb. “Antilocality” effects, in which greater distance between the arguments of a verb result in faster processing time at the verb, suggest that readers preactivate a representation of the verb before encountering it (Vasishth & Lewis, 2006; see Vasishth, 2010, for a review). Bhatia and Dillon (2022) further demonstrate that Hindi comprehenders actively identify and encode an argument NP as agreement controller. Upon encountering an object NP, Hindi and Nepali comprehenders must both identify the morphosyntactic form of the object NP and integrate into a syntactic parse and semantic interpretation of the sentence. As a subprocess, they must identify the morphosyntactic features of the head noun (person, number, and gender features and case inflection) and then access the head noun’s lexical semantics. We assume that these processes are not specific to either language. Brain activity during this period may reflect differences in terms of the morphological form of accusative objects vs. bare objects due to the visual and linguistic form of the object NP, its effect on the entire sentence (e.g., predictability of the verb, construction of a discourse interpration), or differences in interference between the subject and object NPs. Crucially, Hindi comprehenders must then identify the expected agreement controller of the verb according to different principles than Nepali comprehenders. In processing object agreement structures (ergative subject NP, bare object NP sequences—NP-*ne* NP), Hindi comprehenders must represent the object NP as the agreement controller by encoding it in memory with a feature that may be used as a cue for memory retrieval later at the verb (i.e., [Controller]; Bhatia & Dillon, 2022). Furthermore, assuming that agreement processing involves active encoding of expected verb features (Eberhard et al., 2005; Keshev et al., 2025; Keshev & Meltzer-Asscher, 2024), we hypothesize that comprehenders encode an (expected) form of the verb, including its gender, number, and inflectional features, and its aspect (Choudhary et al., 2009). Importantly, the identification of an agreement controller and commitment to the form of the verb presumably happens in Nepali, but it does not need to be time-locked to the processing of the object NP’s morphological features given the irrelevance of the object NP to agreement processing.

## METHODS

### Participants

Twenty-five self-identified native users of Hindi and 24 self-identified native users of Nepali were recruited from the Abu Dhabi community. The Hindi-speaking participants were 19–42 years old (mean = 28, *SE* = 1.4), and the Nepali-speaking participants were 18–42 years old (mean = 24, *SE* = 1.4). All participants had normal or corrected-to-normal vision, and all participants were right-handed except for one Hindi-speaking participant. The Hindi-speaking participants were 7 females and 17 males, and the Nepali-speaking participants were 6 females and 18 males. The study was formally approved by the institutional review board of New York University Abu Dhabi, and all participants gave written consent. Of the Hindi native users, one self-identified as a native user of Spanish, six of English, one of Pashto, two of Gujarati, and two of Nepali. Of the Nepali native users, three self-identified also as native users of Hindi and one of Doteli. Three Hindi-speaking and two Nepali-speaking participants were excluded from analysis due to low signal-to-noise ratio in the MEG sensor data.

### Materials

We prepared 50 sets of items in both Hindi and Nepali. Each item set consisted of eight simple transitive sentences, in SOV word order, written in the Devanagari script. We manipulated the subject case (nominative/ergative), object case (bare/accusative), and verb stem cloze probability (high/low), yielding a 2 × 2 × 2 design in both languages. These are exemplified in Table 1.

**Table 1.**
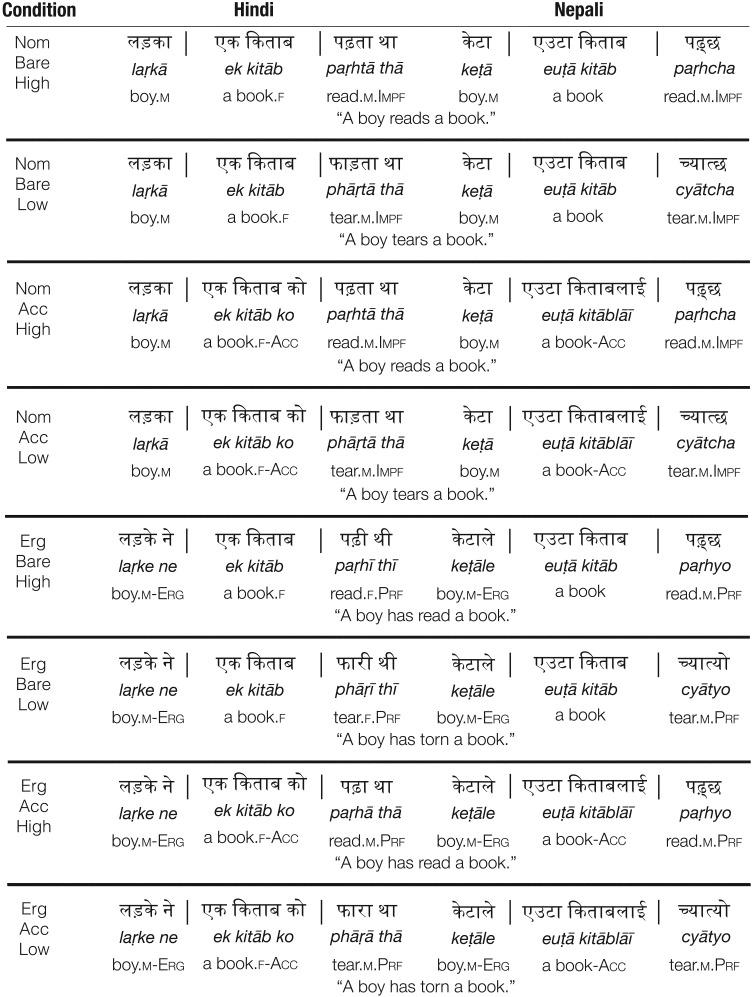
Example stimuli for the experiment, including text in Devanagari script and gloss.

*Note*. Pipes (|) demarcate the three presentation regions: subject NP, object NP, and verb. Gloss abbreviations: Erg = ergative case suffix; Acc = accusative case suffix; m = masculine gender; f = feminine gender; Prf = perfective aspect; Impf = imperfective aspect.

The subject NPs were animate nouns, and the object NPs were inanimate nouns. This was done to ensure that the intended thematic relations between NPs were easily determinable, to ensure both bare and accusative object NPs were grammatically well formed and identifiable, and to make object–subject–verb analyses less likely. The genders of the NPs were counterbalanced, such that the subjects were grammatically masculine in 50% of the items and grammatically feminine in the other 50%. In Hindi, inanimate NPs are also marked for gender, so we ensured that the object NP and the subject NP had opposite gender marking. In Nepali, inanimate NPs are not grammatically marked for gender and default to masculine. Thus, in Nepali, we counterbalanced the gender of the subject NP only.

The verb forms were grammatical in all trials. In Hindi, the verbs were marked with the past perfective endings if the subject was ergative and with the past imperfective endings if the subject was nominative. In Hindi, tense surfaces as a separate auxiliary form, and aspect is marked with a verb stem suffix. Both tense and aspect markers agree with the subject in gender. In Nepali, the verbs were marked with the present perfective endings if the subject was ergative and with the present imperfective endings if the subject was nominative. Due to orthographic conventions, the ergative and accusative case suffixes were written after a space in Hindi and were written connected to the noun stem in Nepali. Because some masculine nouns in Hindi have the same form for the singular and plural, we also included the singular indefinite article एक *ek* “one” before the object NP in Hindi and the singular indefinite article एउटा *euṭā* “one” in Nepali to maintain parallelism between the languages.

Verb stems were chosen to ensure that the sentences all contained plausible thematic relations between the arguments and the verb. However, we varied the predictability of the verb stem as a factor in our design. This M350/N400 manipulation was included as a verification of the design to ensure that both participant groups attended to the stimuli and that we could replicate an otherwise well-attested neural response. We therefore expected to see a similar response that distinguishes between high-probability and low-probability verb stems around 200–400 ms after verb onset in the left temporal lobe for both languages. We treat cloze probability as a binary factor, with levels high cloze probability and low cloze probability.

Verb stem probability was estimated from an online sentence completion task (*N* = 76 for Hindi, *N* = 48 for Nepali). Subject and object NPs were presented in each case combination, distributed in a Latin square design for an internet-based task. Participants were instructed to type a completion for the prompt in either Devanagari or Roman script. Verb stems were identified by two linguists, one a native user of both Hindi and Nepali. The average high cloze probability stems were produced 38.1% ± 3.0% of the time for Hindi and 36.7% ± 2.5% of the time for Nepali, and the average low cloze probability stems were produced in 5.2% ± 0.1% and 3.7% ± 0.8% of the completions for Hindi and Nepali, respectively.

### Procedure

Participants were instructed about the task and signed a consent form electronically. After the participants arrived, we digitized the participant’s head shape using a Polhemus FastScan II (Colchester, VA, USA). This digitized head shape was reduced to a basic surface for use in coregistration with MEG sensor data and magnetic resonance imaging (MRI) structural images. This included eight fiducial points; three points on the forehead and two preauricular points approximately 1 cm in front of the tragus for coregistration with MEG data, and the two tragus points and one nasion point for coregistration with MRI structurals.

After head shape digitization, participants laid supine in a dimly lit, magnetically shielded room (Vacuumschmelze, Hanover, Germany). Participants read sentences in Hindi or Nepali presented phrase-by-phrase (subject NP, object NP, verb) on a projected screen. The stimuli were presented in Devanagari script in the Devanagari MT font, printed in 36 point white font against a dark gray background. Each phrase was displayed for 900 ms, followed by a 100-ms blank screen before the next phrase. This presentation time is longer than in previous studies using Devanagari script (Nevins et al., 2007: 400 + 200 ms; Bickel et al., 2015: 650 + 100 ms), which in turn are longer than typical rapid serial visual presentation paradigms (e.g., 300 + 300 ms). We selected this longer presentation time because the phrases displayed on the screen spanned between one and three orthographic words. The interstimulus interval of 900 + 100 ms was selected as reasonable after pilot runs with the native-speaking authors.

**Figure 2.**
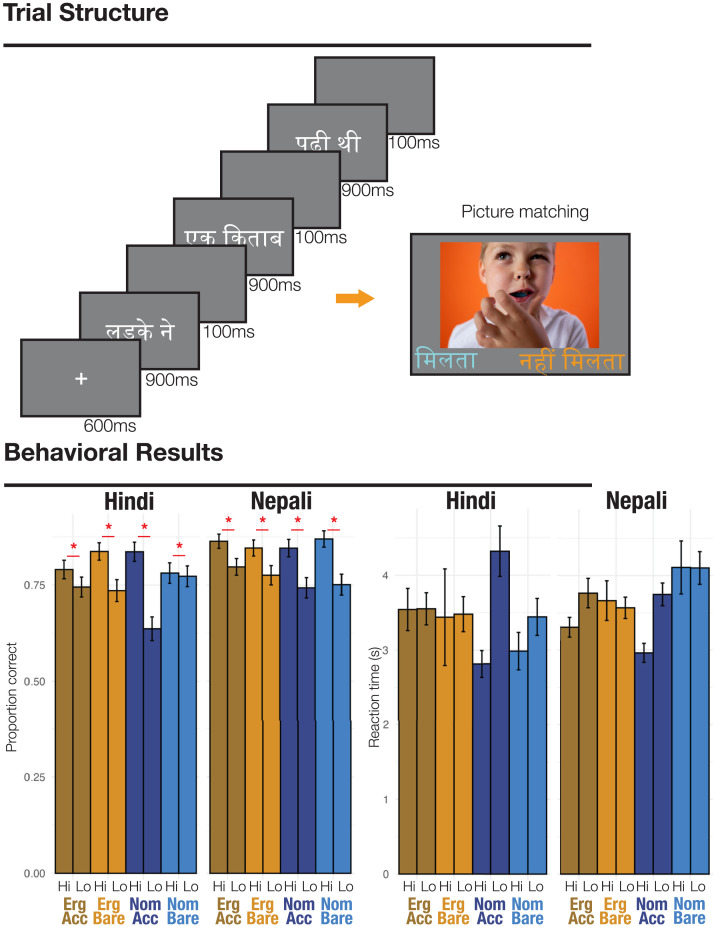
(Top) Task structure. Participants saw a subject NP, object NP, and verb for 900 ms each, followed by 100 ms of blank screen. Then, they responded whether an image matched the sentence they just read by pressing a key on a keyboard. (Bottom) Raw accuracy and reaction time by means by condition. Error bars correspond to one standard error above and below the mean.

Each sentence was preceded by a fixation cross that lasted for 600 ms. After 25% of trials, participants saw a stock photo image and were instructed to indicate whether the stock photo corresponded to the previous sentence using a button box. The stock photos were retrieved from pexels.com and were selected by the authors to either clearly depict the activity described in the photo for the “match” trials or clearly depict a different kind of activity for the “mismatch” trials. The photos were high-definition, full-color photos and were stretched to fill the entirety of the screen. “Mismatch” trials involved either clear depiction of the subject NP referent engaging in a different activity than the one described by the verb phrase, a clear depiction of a different agent referent engaging in the activity described by the verb phrase, or a picture of a different agent engaged in a different activity. For instance, the mismatch trial for sentences corresponding to “the boy read/tore a book” showed an image of a male child eating a lollipop. Thus, participants needed to comprehend both subject NP and the verb phrase in order to succeed on the task. No feedback was provided to participants. Stimuli were presented in a fully within-subject design; each participant saw each trial once. Presentation order was pseudo-randomized in eight blocks, such that each item set appeared once in one condition per block and such that the number of conditions were the same per block. Participants were instructed to take a short break between each block, and the experimenter communicated with the participant during each break. Trial structure is exemplified in Figure 2. All experimental procedures and communication with subjects were conducted in Standard Hindi, Nepali, or English, as per the participant’s request. The experiment lasted approximately 90 min.

Brain signals were recorded using a whole-head 208 axial gradiometer (Kanazawa Institute of Technology, Nonoichi, Japan) at a rate of 1000 Hz with a 0.1-Hz high-pass filter and a 200-Hz low-pass filter applied online. The participant’s head position was measured before and after the experiment using the electrical marker coils attached to the two preauricular and three forehead fiducial points.

### Data Preprocessing

MEG recordings were noise reduced with the CALM method using the MEG160 software (Adachi et al., 2001). All subsequent data analysis was done in MNE-Python (Gramfort et al., 2013). The data were off-line low-pass filtered at 40 Hz using the default settings in MNE-Python. We then used independent component analysis to remove periodic neuromagnetic artifacts, including heart beats, saccades, and eye blinks, and interpolated noisy and flat sensors. We then epoched the sensor data from 200 ms prior to the first phrase to the end of the sentence. We rejected all epochs that exceeded a 5-pT peak-to-peak threshold. Baseline correction was applied using the 200-ms prestimulus period as baseline.

We then projected the data from sensor space to source space. MEG data were coregistered with either a native T1 MRI structural image (*N* = 12 for Hindi, *N* = 7 for Nepali) or the FreeSurfer template brain “fsaverage” (CorTechs Labs Inc., California, USA, and 175 MGH/HMS/MIT Athinoula A. Martinos Center for Biomedical Imaging, Massachusetts, USA). For participants without a native MRI structural image, the fsaverage template brain was scaled in three dimensions to match the digitized headshape and fiducial markers. An ico-4 source space was then built with 2,562 vertices per hemisphere. A forward solution was then computed using the boundary element model (Mosher et al., 1999). Channel noise covariance matrices were estimated using the 200-ms baseline period before all trials and regularized using the automated method (Engemann & Gramfort, 2015). Combining the forward solution and the noise covariance matrices, an inverse solution was computed for each condition for each subject using minimum norm estimate (MNE). For computing the inverse solution, we used a loose dipole orientation with parameter 0.2, yielding signed values. An inverse solution with loose orientation fits a dipole that is orthogonal to the cortical surface but allows the orientation to vary in position to find the best fit (Lin et al., 2006). This inverse solution was selected because not all participants had native MRI structural images, and thus, a fixed orientation may have selected inappropriate orientations for participants that did not have a native MRI structural image. The inverse solution produced a dynamic statistical parameter map (Dale et al., 2000). For group-level analyses and identification of regions of interest, participant’s source spaces were warped to a common fsaverage source space, and anatomical regions were referenced using the parcellation atlases provided by fsaverage brain. For all first-stage regressions, the source-space data were kept as a vector. For subsequent averaging across raw activity in a given spatial region, we used the “mean_flip” function in MNE-Python, which changes the sign of vectors that diverge more than 90° from the dominant direction of the region before averaging across sources.

### Data Analysis

**Figure 3.**
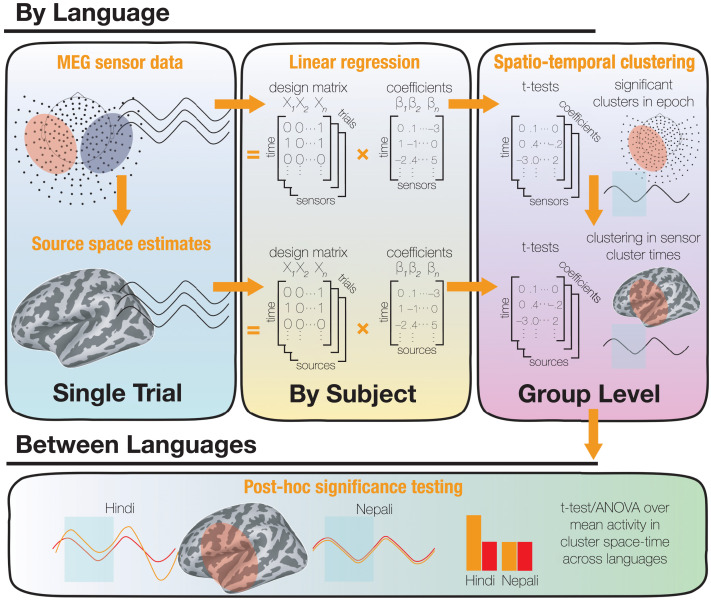
Illustration of steps in the analysis. First, MEG sensor data were projected to source space. Linear models were fit across all trials for each participant for both sensor- and source-space data. Spatiotemporal cluster-based permutation tests were conducted over the resulting beta values in sensor space to identify temporal “windows” of analysis, and source-space analyses were conducted within these time windows in key left-hemisphere language areas. Finally, analyses of variance and *t* tests were conducted over the raw activation in the spatiotemporal coordinates of the source-space clusters for post hoc between-language significance testing.

For data analysis, we chose to use a conservative exploratory approach, prioritizing identifying robust effects in sensor space, followed by temporally constrained analyses in source-space. These analyses were conducted within each language group independently. Afterward, we compared averaged activity from source-space results in each language for across-language comparisons. The analysis pipeline is illustrated in Figure 3.

For each participant, we fit a linear regression to each time point and each spatial coordinate (MEG sensor for sensor-space analyses; source point for source-space analyses) across trials. Sensor-space data were downsampled to 250 Hz prior to linear regression for computational performance reasons. Subject, object, and verb epochs were analyzed independently because different factors were relevant during each of these epochs. During the subject NP epoch, we included the factors subject case, with ergative coded as 1 and nominative/“bare” subject encoded as 0. We also included nuisance regressors of trial number and word length. Trial number was included as a regressor because participants saw the same lexicalizations multiple times throughout the course of the experiment due to the within-subject design, and word length was included because word length is confounded with case marking—adding case suffixes increases word length. During the object epoch, the linear regressions included the same factors but also included object case and subject case: object case interactions. Object case was coded as 1 for bare objects and 0 for accusatives. This coding scheme was selected to ensure that the subject case: Object case interaction term would be coded 1 for ergative subject NP, object NP (NP-*ne* NP) sequences in Hindi, which are specifically predicted to show divergent activity. We also included in the object NP epoch a regressor for the (expected) gender marking on the verb, verb gender. For Hindi, this factor is determined in conjunction with the case marking on both arguments of the verb and their gender specifications, and it is coextensive with the subject NP’s gender given the different syntactic rules of the two languages. The specification of the factor verb gender is given in Table 2. This regressor was included in case we identified divergent responses for sentences that cue an expectation for a verb inflected for masculine vs. a feminine agreement. This factor was coded as 1 for feminine and 0 for masculine. For the verb epoch, we included the same factors as the subject NP epoch because the choice of subject case was coextensive with the form of the verb (ergative case corresponding to perfective aspect; nominative case corresponding to imperfective aspect). We also included the factor verb gender and the factor of verb stem cloze probability, with high coded as 0 and low coded as 1.

**Table 2.** Assignment of the feature verb gender in Hindi and Nepali, as a function of the language, the genders of the subject and object, the subject case, and the object case.

Language	NP genders (subject/object)	Subject case	Object case	Verb gender
Hindi	Masculine subject/feminine object	Nominative	Bare	Masculine
Hindi	Masculine subject/feminine object	Nominative	Accusative	Masculine
Hindi	Masculine subject/feminine object	Ergative	Bare	Feminine
Hindi	Masculine subject/feminine object	Ergative	Accusative	Masculine (default)
Hindi	Feminine subject/masculine object	Nominative	Bare	Feminine
Hindi	Feminine subject/masculine object	Nominative	Accusative	Feminine
Hindi	Feminine subject/masculine object	Ergative	Bare	Masculine
Hindi	Feminine subject/masculine object	Ergative	Accusative	Masculine (default)
Nepali	Masculine subject	Nominative	Bare	Masculine
Nepali	Masculine subject	Nominative	Accusative	Masculine
Nepali	Masculine subject	Ergative	Bare	Masculine
Nepali	Masculine subject	Ergative	Accusative	Masculine
Nepali	Feminine subject	Nominative	Bare	Feminine
Nepali	Feminine subject	Nominative	Accusative	Feminine
Nepali	Feminine subject	Ergative	Bare	Feminine
Nepali	Feminine subject	Ergative	Accusative	Feminine

After fitting the regressions for each participant in sensor and source space for each epoch, we then conducted one-sample *t* tests across the participant’s beta coefficients for each theoretically relevant predictor at each spatiotemporal point to determine whether the betas were significantly different than 0. We then conducted spatiotemporal cluster-based permutation tests to evaluate the *p* value and correct for multiple comparisons (Maris & Oostenveld, 2007). First, we identified adjacent spatiotemporal points that were identified as significant with an uncorrected threshold of *p* ≤ 0.05. Then, we randomly replaced beta values with 0 and repeated this clustering procedure 10,000 times to produce a null distribution of clusters. Cluster “sizes” were calculated by summing the *t* values and ordered by size. The attested clusters were considered significant at *p* ≤ 0.05 if they were in the extreme 2.5% tails of this null distribtution, or marginally significant at *p* ≤ 0.10 if they were in the extreme 5.0% tails of this null distribution.

Spatiotemporal cluster-based permutations were initially conducted in sensor space across the entire length of each time span (0–1,000 ms). For the verb epoch, we instead selected a more conservative 300–700 ms time span, since we expected a typical “M350” response, ∼200–400 ms post–verb onset. This analysis time window is a bit later and more extended than might be typical, but visual inspection of the sensor-space data suggested that the neural responses were more diffuse in space in both Hindi and Nepali populations. This may have been because the “verb” epoch included one or two orthographic words depending on the language and condition.

The results of the sensor-space analyses were then taken as “temporal localizers” of key effects, and then the spatiotemporal cluster-based permutation tests were conducted in source-space as a secondary exploratory analysis. These spatiotemporal cluster-based permutation tests were conducted in a left-hemisphere “language network” mask, including the *aparc* labels included in the fsaverage template brain (Desikan et al., 2006) for the inferior frontal gyrus (pars orbitalis, pars triangularis, pars opercularis), the insula, the superior and middle temporal gyri, the transverse temporal gyri, the banks of the superior temporal sulci, and the supramarginal and angular gyri. After the time-constrained analyses in the left-hemisphere perisylvian regions, we then conducted exporatory analyses during the entire epoch (0–1,000 ms) in the whole brain using a *p*-value threshold of *p* ≤ 0.10.

After identifying clusters in the source-space spatiotemporal analysis, we then computed the averages of activity in both the Hindi and Nepali populations from this region, taking the maximum dipole value per spatiotemporal coordinate per participant. We then conducted post hoc pairwise *t* tests comparing participant activations in this region between the target ergative subject NP, bare object NP conditions (object agreement constructions in Hindi) and the nominative subject NP, bare object NP conditions, and similarly between the accusative object NP trials nested within the factor of subject case. These pairwise *t* tests test the hypothesis that neural activity in this identified region of interest reflects different activity for object agreement constructions in Hindi that is not observed in Nepali or for accusative object NP configurations in either language. The steps of this analysis pipeline are illustrated in Figure 3.

## RESULTS

### Behavioral Results

For behavioral results, we fit a logistic linear mixed-effects model in R to the accuracy and another to the reaction times on the trials with the picture-matching task (Bates et al., 2015; R Core Team, 2021). In both analyses, we fit one model to data from both languages. Each model was fit with fixed effects for subject case, object case, verb stem cloze probability, and their interaction terms. These factors were sum-coded, with 1 for ergative, accusative, and low cloze probability, respectively, and −1 for nominative, bare, high cloze probability. We also included a fixed effect of language, with Hindi coded as 1 and Nepali as −1.

We initially fit a “maximal” random effect structure (Barr et al., 2013), including random slopes for all fixed effects by participant and by item. These models did not initially converge, so we used backward elimination of terms in the random effect structure using likelihood ratio tests until the model converged. In all cases, this procedure resulted in a model with random intercepts for participant and item only. The summary of these findings are presented in Table 3.

**Table 3.** Results of mixed-effects models fit to the reaction time and accuracy for the picture matching task.

**Reaction time**	***β***	***SE***	***t***	***p***
(Intercept)	3628.7	277	13.1	**<0.01**
Subject case	−64.7	113.4	−0.56	0.58
Object case	−0.4	113.4	−0.03	1.0
Cloze	−195.1	63.4	−3.1	**<0.01**
Language	−62.1	168.5	−0.37	0.71
Subj:Obj	−35.0	113.4	−0.31	0.76
Subj:Cloze	−143.0	63.4	−2.3	**0.02**
Obj:Cloze	−134.4	63.4	−2.1	**0.03**
Subj:Obj:Cloze	−76.4	63.4	−1.2	0.23

**Accuracy**	***β***	***SE***	***t***	***p***
(Intercept)	1.74	0.18	13.1	**<0.01**
Subject case	−0.03	0.12	−0.56	0.79
Object case	−0.05	0.12	−0.03	0.67
Cloze	0.38	0.04	−3.1	**<0.01**
Language	−0.08	0.15	−0.37	0.60
Subj:Obj	−0.05	0.12	−0.31	0.68
Subj:Cloze	0.03	0.04	−2.3	0.43
Obj:Cloze	0.04	0.04	−2.1	0.33
Subj:Obj:Cloze	0.12	0.04	2.7	**<0.01**

*Note*. The structure of each model was: Correct/Reaction Time ∼ Subject Case * Object Case * Verb Cloze Probability + Language + (1|Participant) + (1|Item). **Bold** indicates *p* values of < 0.05. Units are ms (reaction time model) and log odds probability (accuracy model).

For both Hindi and Nepali participants, responses were more accurate and faster to sentences with high-probability verbs compared to low-probability verbs (Reaction Times: *β* = −390, *SE* = 127, *z*-ratio = −3.1, *p* < 0.01; Accuracy: *β* = 0.76, *SE* = 0.09, *z*-ratio = 8.5, *p* < 0.01). We also observed the reaction time model two-way interactions between verb stem cloze probability and both subject case and object case, and in the accuracy model, a three-way interaction between subject case, object case, and verb stem cloze probability. Post hoc pairwise comparisons revealed that high-probability verbs were read more quickly than low-probability verbs after nominative subject NPs (*β* = −676, *SE* = 176, *z* ratio = −3.8, *p* < 0.01) and after accusative object NPs (*β* = −659, *SE* = 186, *z* ratio = −3.4, *p* < 0.01) and were responded to more quickly after all case combinations (all *p*s < 0.01). The mean acceptability and response times are shown in Figure 2.

### MEG Results

#### Verb epoch analyses

We predicted that MEG activity should diverge between the high- and low-stem cloze probability verb stems approximately ∼300 ms in both Hindi and Nepali, reflecting a typical “M350”/“N400” effect for an unexpected word.

**Figure 4.**
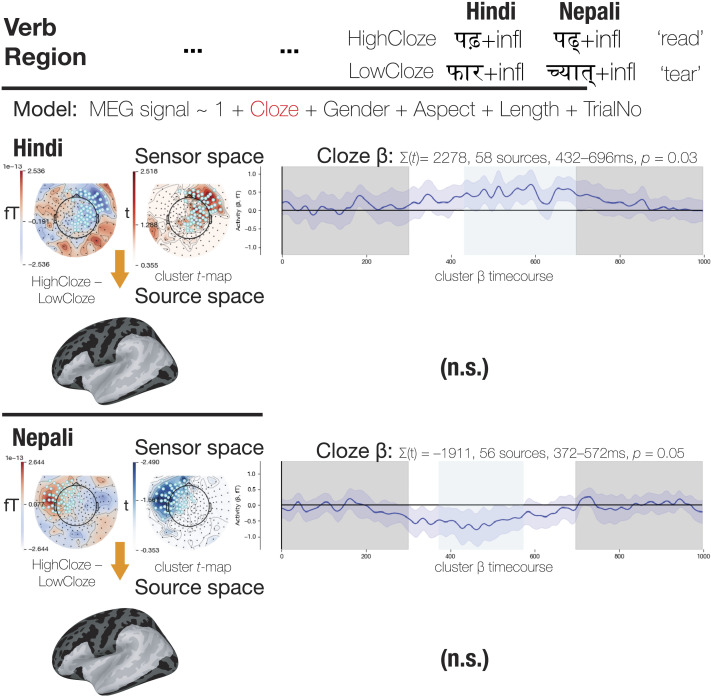
Results of whole-epoch sensor-space analysis during the verb period in Hindi and Nepali. Topographic plot shows the distribution of the thresholded significant *t* values in the cluster and the difference topoplot between the accusative and bare conditions. The time course shows the average activity of these sensors, with temporal extent of cluster highlighted in blue.

Sensor-space analyses in Hindi revealed a positive cluster for verb stem cloze probability in right anterior sensors from 432 to 696 ms (58 sources, *p* = 0.03, Σ(*t*) = 2278), and sensor-space analyses in Nepali revealed a negative cluster for verb stem cloze probability in left anterior sensors from 372 to 572 ms (56 hr, *p* = 0.05, Σ(*t*) = −1911). Topographic maps revealed a similar bipolar magnetic field in both language groups, with a greater positive magnetic field in the left anterior regions and a greater negative magnetic field in the right anterior regions, consistent with an anterior midline field distribution (Pylkkänen & McElree, 2007; see Pylkkänen et al., 2009, for review; see Figure 4).

#### Object epoch analyses

The goal of this analysis was to identify a neural response sensitive to the case marking of the object NP. We predicted that this neural response would show an interaction between the case marking in Hindi, corresponding to the identification of the object NP as an agreement controller in the ergative subject, bare object (NP-*ne* NP) condition, and that this expected neural response would not be observed in Nepali. To this end, we conduct the sensor- and source-space analysis as described in the Data Analysis section, identifying clusters in time corresponding to object NP case features, followed by source-space analyses to identify potential neural generators, followed by between-language comparisons of the averaged raw activity in the cluster.

**Figure 5.**
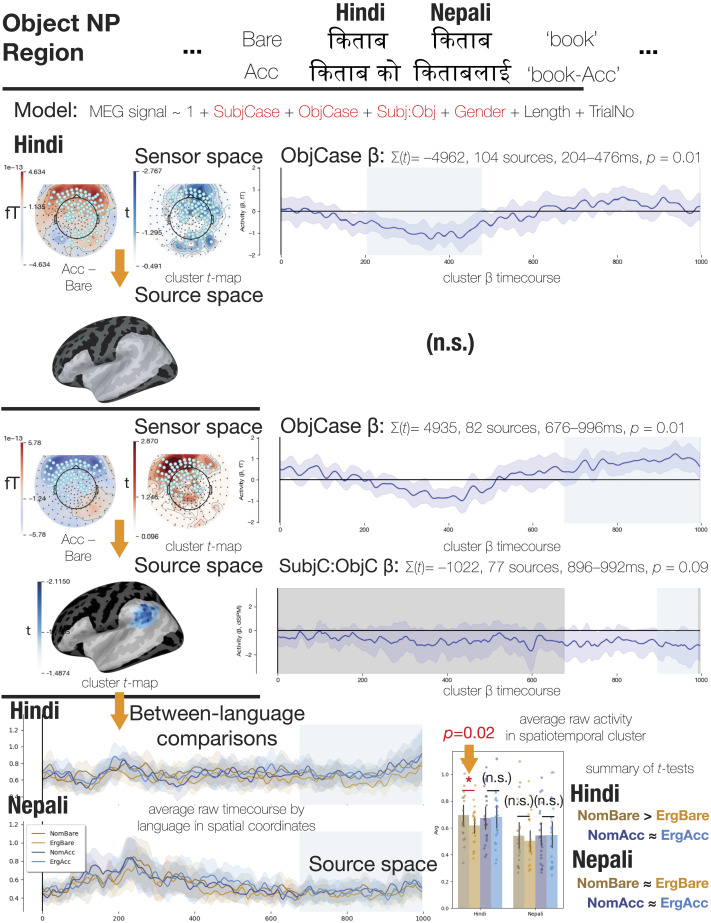
Results of whole-epoch sensor-space analysis during the object NP period in Hindi. Topographic plot shows the distribution of the thresholded significant *t* values in the cluster and the difference topoplot between the accusative and bare conditions. The time course shows the average activity of these sensors, with temporal extent of cluster highlighted in blue. Brain plot shows the distribution of *t* values in the subsequent source-space analysis conducted within a left-hemisphere perisylvian mask (highlighted in white), constrained within the time points of the sensor-space cluster. Raw activity in this source-space cluster are plotted for both Hindi and Nepali participants. Bar plots show the by-subject average activity in space-time, and reported *p* values correspond to pairwise *t* tests between accusative and bare levels embedded within each factor of subject case, by language.

In the sensor-space analyses during the object NP epoch, we identified two significant clusters in Hindi and Nepali for object case. In Hindi, we observed a negative cluster over central anterior and right posterior sensors, from 204 to 476 ms post–object NP onset (104 sources, *p* = 0.01, Σ(*t*) = −4962), and a positive cluster over left and central anterior sensors, from 676 to 996 ms (82 sources, *p* = 0.01, Σ(*t*) = 4,935). These effects correspond to a greater positive magnetic field and a greater negative magnetic field in frontal sensors for accusative object NPs, respectively. No significant clusters were observed in the left-hemisphere regions during the 204–476 ms period (*p*s > 0.10). During the 676–996 ms period, we observed a marginally significant cluster for the Object Case × Subject Case interaction. This cluster was located in the supramarginal gyrus in the LTPJ during the end of this time period, 896–992 ms (77 sources, *p* = 0.09, Σ(t) = −1,022). Post hoc comparisons of the raw activity by condition in these spatial coordinates in Hindi and Nepali revealed a similar time course, with a peak around ∼200 ms, characteristic of MEG signal recorded from LTPJ. During the 896–992 ms period, there was a reduction in activity for the object agreement configurations in Hindi, i.e., the ergative subject NP, bare object NP (NP-*ne* NP) configurations. Pairwise *t* tests demonstrated that the averaged activity in these spatiotemporal coordinates were greater for nominative subject NP, bare object NP conditions (subject agreement) configurations than the ergative subject NP, bare object NP constructions (object agreement) in Hindi (*p* = 0.02), but not in Nepali (*p* > 0.10). Similarly, the difference between the bare object NP conditions and the accusative object NP conditions nested within each level of the factor subject NP were not significant in Nepali (*p*s > 0.10). These results are shown in Figure 5.

**Figure 6.**
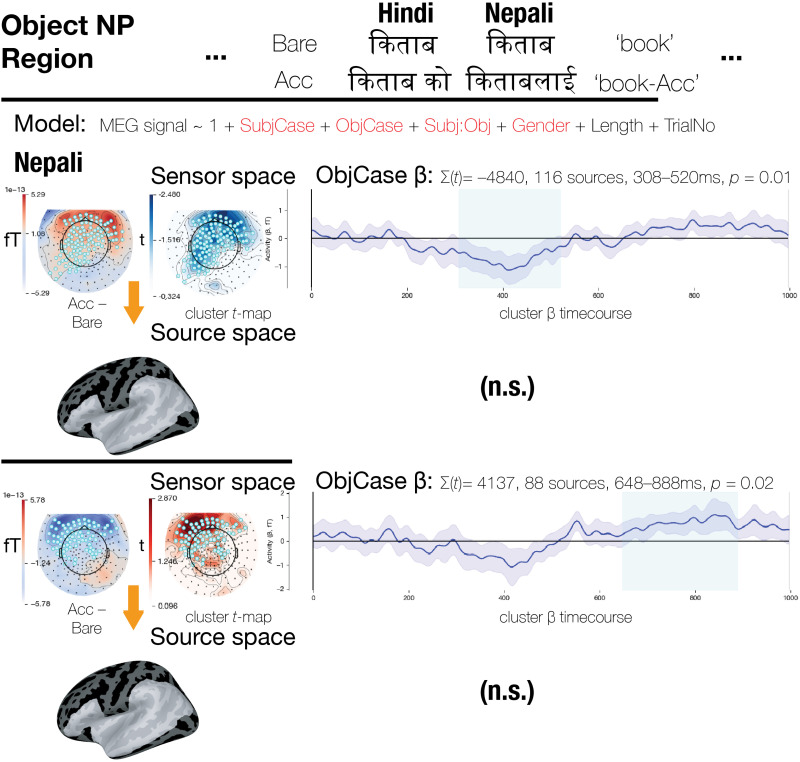
Results of whole-epoch sensor-space analysis during the object NP period in Nepali. Topographic plot shows the distribution of the thresholded significant *t* values in the cluster and the difference topoplot between the accusative and bare conditions. The time course shows the average activity of these sensors, with temporal extent of cluster highlighted in blue.

In sensor-space analyses in Nepali, we also observed two significant clusters of object case. The first was a negative cluster observed in central anterior and left parietal sensors, from 308 to 520 ms (116 sources, *p* = 0.01, Σ(*t*) = −4,840). The second was a positive cluster over left and central anterior sensors, from 648 to 888 ms (82 sources, *p* = 0.02, Σ(*t*) = 4,137). As with Hindi, inspection of the topographic maps shows a greater positive magnetic field for accusative marked object NPs during the earlier period and a greater positive negative magnetic field for accusative marked object NPs during the later period. Source-space analyses constrained to these time windows did not yield any significant clusters in Nepali (*p*s > 0.10). These results are shown in Figure 6.

#### Subject epoch analyses

Analyses conducted during the subject NP epoch were exploratory analyses intended to identify whether there were different neural correlates for ergative subject NPs vs. bare subject NPs in either Hindi or Nepali.

**Figure 7.**
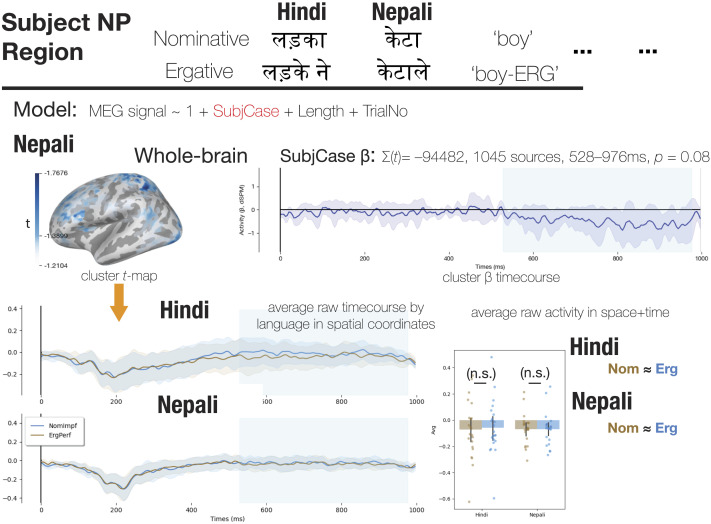
Results of whole-brain exploratory analysis during the subject NP period in Nepali. Brain plot shows the distribution of the thresholded significant *t* values in the cluster, and time course shows the average activity of these sources, with temporal extent of cluster highlighted in blue. Raw activity in this cluster are plotted for both Hindi and Nepali participants. Bar plots show the by-subject average activity in space-time.

Sensor-space analyses did not reveal any significant clusters of subject case in either Hindi or Nepali (*p*s > 0.10). Exploratory whole-brain analyses throughout the 1,000-ms epoch also revealed no significant clusters in Hindi (*p*s > 0.10). In Nepali, we identified one marginally significant cluster that spanned the left hemisphere. Because this cluster spanned the majority of the left hemisphere (2,471/2,562 sources), we thresholded this cluster to source points with averaged absolute values of *t* ≥ 1.2. This cluster spanned posterior temporal lobe, middle and inferior parietal lobe, and middle and inferior frontal lobe, from 528 to 976 ms post–subject NP onset (1,045 sources, *p* = 0.08, Σ(*t*) = −94,482). Post hoc comparisons of raw activation in these spatial coordinates did not reveal a significant difference between the ergative and nominative sentences (*p*s > 0.10), but a sustained negativity was observed in the time course of the Hindi data during this period. These results are shown in Figure 7.

## DISCUSSION

In both Hindi and Nepali, we observed that MEG responses showed a reduction in the anterior midline field response ∼350–500 ms for low cloze probability verb stems. We also found that bare object NPs in both Hindi and Nepali elicited a reduction in an anterior positive field ∼300–500 ms and a reduction in an anterior negative field ∼600–900 ms post–object NP onset. This demonstrates similar neural mechanisms involved in processing both NP case and verbs. An exploratory analysis in Nepali revealed a distributed response for ergative subject NPs ∼500–1,000 ms distributed throughout the left hemisphere, including the LIFG and LPTL regions.

Most critically for the research question, we did not observe a reliable difference between the brain activity associated with case marking in Hindi or Nepali during the processing of the object NP.

### Differences in Case Processing in Hindi and Nepali

The primary research question pursued in this article is whether the grammatical differences between Hindi and Nepali with respect to the interactions between case marking and agreement would be reflected in the MEG signal evoked by an agreement controlling NP. Previous electrophysiological and behavioral psycholinguistic research in Hindi suggests that comprehenders use case marking to make early commitments to the form of the verb and actively identify an NP as the controller for agreement (Bhatia & Dillon, 2022; Bhattamishra et al., 2022; Choudhary et al., 2009). We therefore predicted that neural activity elicited during the processing of an object NP in a sentence that required object–verb agreement should differ systematically than an object NP in a sentence that does not, either in the same language or a different language.

We failed to find evidence for this hypothesis. Instead, we observed a similar biphasic pattern, showing a reduction in the amplitude of an anterior positive field ∼300–500 ms and a reduction in the amplitude of an anterior negative field ∼500–900 ms after an object NP was encountered. No planned analysis revealed a predicted interaction effect of case marking in Hindi. Thus, the data suggest that case marking is processed in similar fashion between the two languages, even though in Hindi this information is crucial for identifying an agreement controller and encoding an expectation for the verb’s agreement features.

Despite this, in subsequent analyses in the temporal coordinates of the latter component of the biphasic response to object case, we observed a nonsignificant cluster (*p* = 0.09) for the Subject Case × Object Case interaction in Hindi located in the supramarginal portion of the LTPJ. Subsequent between-language group-level analyses on raw activity from this region during this time window show a difference between the object agreement conditions (ergative subject NP, bare object NP) and the minimally matched subject agreement control (nominative subject NP, bare object NP) in Hindi, but not Nepali.

Although we cannot draw firm conclusions about differences between the cognitive processes deployed in processing an agreement-controlling NP vs. a non–agreement-controlling NP or between users of Hindi and Nepali, we highlight that the LTPJ may be a useful “hotspot” for attention in future research related to integration of syntactic cues in anticipatory structure-building processes, specifically with regard to similarities and differences in processing argument–verb agreement relations.

The LTPJ and its right-hemisphere homolog appear to have a wide range of functions (Seghier, 2013). In language processing, the TPJ is usually thought to be key to the understanding of event representations (Binder et al., 2009; Binder & Desai, 2011), relational semantic or thematic relations (Lewis et al., 2015; Thompson et al., 2007; Thothathiri et al., 2012; Williams et al., 2017), semantic composition (Boylan et al., 2015; Zhang et al., 2022), and to making logical inferences (Xu et al., 2024). Other work suggests that the TPJ may play a key role in processing of relations across sentences in discourse (Kandylaki et al., 2016; Tulling et al., 2021). Finally, the TPJ is implicated in a wide array of broader cognitive operations involving theory of mind, perspective taking, and self–other distinctions (Koster-Hale et al., 2017; Quesques & Brass, 2019; Saxe & Wexler, 2005).

One possible interpretation of our results is that the LTPJ response reported in Hindi is an anticipatory response prior to encountering the verb. In a combined fMRI-MEG study, Matchin et al. (2017) report an increase in LTPJ activity at the closure of a verb phrase, which they attribute to the composition of a semantic representation of an event description (e.g., Binder & Desai, 2011). Hindi and Nepali are both verb-final, so participants may have exploited the semantics of the object NP to predict some aspects of the argument structure prior to the verb. However, it is not clear how this would explain why there’s a reduction for object agreement configurations in Hindi in LTPJ but not in Nepali, given that the thematic relations between the arguments and verbs were virtually identical in the two experiments.

Outside of language processing, bilateral TPJ corresponds to attention shift (Corbetta & Shulman, 1998; Seghier, 2013; Singh-Curry & Husan, 2009), especially attending to task-relevant bottom-up features (Ciaramelli et al., 2008), and may serve a “hub” for integration of processes in the prefrontal regions and posterior parts of the language network (Kim et al., 2022; Seghier, 2013). In an SOV language like Hindi or Nepali, selection of an agreement controller for object agreement constructions can be conceived of as requiring a shift in attention from the subject NP to the object NP. In subject agreement configurations, the grammatical features of the subject NP must be “carried forward” to the verb, whereas in object agreement configurations the features of the subject NP must be suppressed in favor of those of the object NP.

We also note that we did not identify a region in source-space that corresponded to the sensor-space results. There are a few reasons why there may be mismatches between sensor- and source-space results (see Schaworonkow & Nikulin, 2022, for a concrete example). First, the sensor-space analyses we reported here were conducted on raw magnetic field amplitude, whereas source-space analyses are the output of the inverse solution model. The inverse solution fits dipoles relative to the cortical surface across a greater number of sources (2,562 per hemisphere) than the number of sensors in the raw recording (208 sensors). Significance is evaluated by a permutation test, in which a null distribution is bootstrapped from the data across spatiotemporal coordinates, of which there are substantially more of in the source-space analyses. These factors may affect the spatiotemporal profile of an effect in source-space, i.e., sensor-space results might be less detectable with a spatiotemporal cluster-based permutation because they are observed in only a small number of sensors, whereas real experimental effects and bootstrapped clusters in the null distribution may be magnified or artificially “smeared” when projected into source-space. Similarly, MRI structural images were only obtained for some of the participants in both the Hindi and Nepali samples, and thus, some participants’ inverse solutions may have been less precise than others. Second, the forward solution takes into consideration the relative position of the MEG sensors to the participant’s head (and structural MRI image), whereas sensor-space analyses report on the magnetic field signal recorded at each sensor regardless of its position relative to the head in a common space. Minor differences in head sizes, positions, and orientations may have altered the spatial distribution of an effect in sensor-space that is “corrected” in source-space.

However, we do note that the exploratory analysis in the subject NP time window did reveal a nonsignficant distributed response in LIFG, LMFG, LTPJ, and LPTL for ergative-marked subject NPs vs. nominative-marked subject NPs. These regions have been identified in previous hemodynamic and electrophysiological studies on case marking (Bornkessel et al., 2005; Nieuwland et al., 2012; Quiñones et al., 2014, 2018; Yokoyama et al., 2012). As with the LTPJ analysis, we are cautious to interpret these results. This was not observed in the object NP time window analyses and thus may not reflect differences in processing case marking in genera. Moreover, the interpretation of this difference in activity could be the result of several different processes. These differences in responses may reflect the morphosyntactic processing of the noun into its stem and case ending. In both languages, the subjects marked with ergative case imply that the sentence will terminate with a perfective verb, a cue that is rapidly integrated into incremental sentence processing (Choudhary et al., 2009). However, ergative case may also be used in some contexts to signal agentivity or to signal other semantic aspects of the predicate. Thus, activity associated with main effects of either subject or object NP case morphology could in principle reflect aspects of the NP case, its semantic consequences, or its consequences for predictive processing of upcoming material.

### Verb Cloze Probability Effect

The verb cloze probability manipulation was included as an independent verification that participants were attending to the task and processing the sentences in a similar way. We predicted that the neural response would emerge as a typical M350 response 200–400 ms post–verb onset and localize to posterior portions of LMTG, although similar N400/M350 effects may also localize to portions of LIFG, OF, or LTPJ (Lau et al., 2008). This prediction was not borne out. Instead, we observed an anterior midline field from approximately 350 to 700 ms in sensor-space and did not identify a corresponding cluster in source-space. The anterior midline field has been reported for semantic complement coercion effects and semantically anomalous words in context 350–500 ms post–word onset (see Pylkkänen et al., 2004, 2009, for reviews), rather than words that are merely unpredictable.

This delay may have been due to the increased complexity of the stimuli compared to stimuli from other N400 studies. In our experiment, the epochs consisted of multiple phonological/orthographic words that were displayed in parallel, which may have led to a delay in the identifiability of a response corresponding to lexical access of the verb stem. Alternatively, the delayed response may reflect additional complexities introduced by the Devanagari writing system, although this may be unlikely, since other authors have reported typical N400 latencies (Bhattamishra et al., 2022; Choudhary et al., 2009). Another potential explanation is that this delay may have reflected an adaptation to the slower-than-usual presentation speed of 1,000 ms per phrase. Participants may have adjusted their reading strategy or may have withheld attention at the initial presentation of the word and phrase, thereby delaying stages of lexical access compared to the onset of the stimulus. Finally, “N400” responses to unpredictable stimuli may not be neurobiologically distinct from other responses to unpredictable stimuli that otherwise differ in latency or space (LAN, mismatch negativity; Bornkessel-Schlesewsky & Schlesewsky, 2019); thus, an increase in latency compared to the typical time window may not “rule out” considering these responses as a member of the N400 family once these other factors are accounted for.

We also note that the cloze probability of our stimuli was overall lower than is typical in N400 studies that categorically contrast brain activity with cloze probability. High cloze probability stimuli usually have completion rates ∼40%–50%, and low cloze probability stimuli are closer to ∼5%–10%, whereas ours were lower than these. This could have also tempered the robustness of an M350 response that may have otherwise been observed in the N400 time window. Alternatively, although we intended for the low cloze words to be semantically congruous with the arguments, the exceptionally low probability of these verbs may reflect that some proportion of our trials had a semantically anomalous interpretation.

Finally, we note that in both languages, the factor verb stem cloze probability was distributed across different aspect markers and gender agreement. For instance, in Hindi, the verb stem cloze probability factor collapses across both genders and aspects (High: पढ़ा था *paṛhā thā* read.Perf.Masc, पढ़ी थी *paṛhī thī* read.Perf.Fem, पढ़ता था *paṛhtā thā*, read.Impf.Masc, पढ़ती थी read.Impf.Fem; Low: फारा था *phārā thā* tear.Perf.Masc, फारी थी *phārī thī* tear.Perf.Fem, फारता था *phārtā thā* tear.Impf.Masc, फारती थी *phārtī thī* tear.Impf.Fem). We designed the experiment with the assumption that the predictability of the verb stem would be isolatable as an independent factor; it may be in principle that certain verb stems may be more or less likely in specific aspects or with certain gender inflections, or that different combinations of NP case markings may alter the predictability of verb stems. Similarly, if similar neuroanatomical regions are engaged in processing the verb stem as well as its morphosyntactic features, then there may be no distinct, separable effects of the verb stem identifiable in the brain.

Other studies in Hindi have demonstrated negative-going ERPs 200–500 ms in studies contrasting predictable stimuli vs. violations. Choudhary et al. (2009) report on differences in N400 amplitudes for unlikely subject NP case–verb aspect combinations (ergative-imperfective; nominative-perfective). Bhattamishra et al. (2022) report on N400(-like) responses for verb–subject agreement mismatches between inanimate subject NPs and verbs. Thus, other studies have demonstrated N400 effects for formal inflectional features on the verb given the prior arguments. By contrast, our study focused on the prediction of the morphosyntactic features of the verb, but we did not deploy a prediction violation paradigm. How do previous N400 findings in Hindi relate to “traditional” N400 effects, corresponding to more vs. less predictable verb stems? To our knowledge, no previous study has contrasted neural responses the (un)predictability of the verb stem vs. verb inflection directly, and thus, we leave the question of the commensurability of the M350/N400 corresponding to identification of a verb stem and processing of its inflectional features as an open question.

Finally, we observe that the polarity of the clusters reported for the verb stem cloze probability manipulation differs in the statistical results for the Hindi and Nepali analysis. The spatiotemporal cluster-forming procedure was constrained such that *t* values of the same sign were clustered together, and we report only on the largest cluster observed in each analysis. Although we report on a positive cluster in Hindi and a negative cluster in Nepali, we observe that the topographic maps are largely similar in showing an anterior midline field pattern in both languages. Moreover, the positive cluster in Hindi is largely constrained to the negative field, and the negative cluster in Nepali is largely constrained to the positive field. Thus, we take these findings as indexing the same response, although the statistically significant clusters were observed in different sides of the bipolar field.

## CONCLUSION

The human brain is capable of learning and representing any natural human language. Moreover, left frontotemporal and temporoparietal language regions subserve language comprehension across a variety of languages and language users. Yet, different grammatical rules may require that comprehenders store, attend to, or predict different properties of words and phrases, processes that must be supported by the brain. Additionally, users of different languages appear to integrate different cues into their interpretation of a sentence, reflecting different properties of their language. What is the relation between the otherwise uniform “language network,” parametric grammatical differences, and language-specific adaptations that comprehenders exhibit as a function of their specific languages? In general, this is a difficult question to answer, because cross-language comparisons often confound many factors, i.e., very few languages only differ along one parameter of variation.

Here, we present on two parallel MEG experiments in Hindi and Nepali. We exploit similarities between the grammatical rules governing case assignment and differences between the grammatical rules governing verb agreement to determine how the brain encodes and predicts features of an upcoming verb, a key subprocess in sentence processing.

In both Hindi and Nepali, we observed strong differences between case-marked and non–case-marked object NPs in anterior fields from approximately 300–500 and 600–900 ms. We did not observe a statistically significant difference between the two languages, suggesting that the processing of case features is largely similar between users of Hindi and Nepali. Subsequent exploratory analyses identified a nonsignificant effect in portions of the LTPJ that may subserve different functions in Hindi and Nepali. However, we must leave the relevance of this region for the processing of case and agreement for future work. Regardless, our findings validate the utility of carefully controlled, cross-language experiments in the cognitive neuroscience of language and the importance of research in less-studied languages for psycholinguistic models in general.

## ACKNOWLEDGMENTS

The authors would like to thank Liina Pylkkänen, Swarnendu Moitra, and the audiences of Human Sentence Processing, Society for Neurobiology of Language, and Architectures and Mechanisms for Language Processing for useful feedback and insight.

## FUNDING INFORMATION

Alec Marantz, Research Institute Centers, New York University Abu Dhabi (https://dx.doi.org/10.13039/100020770), Award ID: G1001.

## AUTHOR CONTRIBUTIONS

**Dustin Alfonso Chacón**: Conceptualization: Lead; Data curation: Lead; Formal analysis: Lead; Investigation: Lead; Methodology: Lead; Visualization: Lead; Writing – original draft: Lead; Writing – review & editing: Lead. **Subhekshya Shrestha**: Data curation: Equal; Investigation: Equal; Writing – review & editing: Equal. **Brian W. Dillon**: Conceptualization: Supporting; Writing – original draft: Supporting; Writing – review & editing: Supporting. **Rajesh Bhatt**: Data curation: Supporting; Investigation: Supporting; Methodology: Supporting. **Alec Marantz**: Conceptualization: Equal; Funding acquisition: Lead; Supervision: Equal; Writing – review & editing: Equal. **Diogo Almeida**: Conceptualization: Supporting; Funding acquisition: Supporting.

## DATA AND CODE AVAILABILITY STATEMENTS

Preprocessed MEG data and processing code have been made publicly available on Open Science Framework at https://osf.io/qrzpm.

## TECHNICAL TERMS

Ergative case: A morphological case associated with agentive subject NPs and commonly with perfective aspect in “split-ergative” languages.
Bare case: A morphologically unmarked case associated with subject NPs and object NPs in South Asian languages.
(Argument)–verb agreement: Refer to the match in morphological features between a verb and an argument NP in the sentence; e.g., the *boy-ø run-**s*** vs. the *boy-**s** run-ø*.
Accusative case: A morphological case associated with direct object NPs and indirect object NPs.
